# Meeting report

**DOI:** 10.1155/S146392469900019X

**Published:** 1999


- ultrahigh throughput screening and assay miniaturization
- automation technologies for genomics
- secondary screening
- assay optimization and methods development
- data management/data handling and bioinformatics
- introduction to laboratory robotics
- automation strategies for assay development and primary and secondary screening
- dissolution testing: theory, practice and automation techniques
- bioanalytical automation: trends, technology and new developments
- EasyLab® programming: tips and tricks
- PCS™: tips and tricks

The three-day programme consists of over 120 podium and poster presentations describing laboratory robotics and laboratory workstation procedures from pharmaceutical, biotechnology and chemical laboratories. The symposium's technical presentations and informal discussions provide an excellent opportunity for laboratory managers, automation users and novices to exchange ideas and applications experience.

*For more information write or call: Christine O'Neil, ISLAR, 68 Elm Street, Hopkinton, MA 01748, USA. Tel: +1 508 497 2224, Fax: +1 508 435 3439, e-mail: islar@islar.com, URL—http://www.islar.com*

### Meeting report

### ISLAR '98 Recognizes Pioneers in Laboratory Automation and Robotics

The 16th International Symposium on Laboratory Automation and Robotics, held at the Boston Park Plaza Hotel during 18–21 October, recognized six scientists for their roles in developing new applications and techniques, designing unique solutions to technical problems, and implementing laboratory automation in their organizations. Nominated by colleagues in their companies or industry, these scientists were selected for this recognition by a committee of previous award winners.

The six scientists included:

Muhammed Alburakeh, Barr Laboratories, Pomona, NY, USA;
Siegfried Brandtner, Grunenthal GmbH, Germany;
Harnath Doddapaneni, SmithKline Beecham Pharmaceuticals, King of Prussia, PA;
Jim Perry, Procter & Gamble, UK;
Richard Wildonger, Zeneca Pharmaceuticals, Wilmington, DE, USA;
Rudy Willebrords, Janssen Research Foundation, Belgium.

ISLAR '98 attracted about 700 scientists from 33 states and 19 countries for three days of presentations on laboratory automation and robotics. The premier technical symposium in this field featured a program with nearly 150 papers and posters, seven discussion groups and several workshops, and six hands-on short courses, including the following areas of interest.

- High throughput screening.
- Data management/data handling.
- Ultra high-throughput screening.
- Managing laboratory automation.
- Methods transfer/validation.
- Pharmaceutical analysis.
- Bioanalytical applications.
- Advanced topics.
- Automated synthesis.
- Compound purification, distribution and analysis in drug discovery.
- Managing pharmaceutical development and QC laboratories.
- Automated physical testing & chemical analysis.
- Consumer products/food applications.
- Predictive design/structural characterization.

*For more information contact: Christine O'Neil, ISLAR, 68 Elm Street, Hopkinton, MA 01748, USA; Tel.: +1 508/435-9500; Fax.: +1 508/435-3439; e-mail: islar@islar.com*

